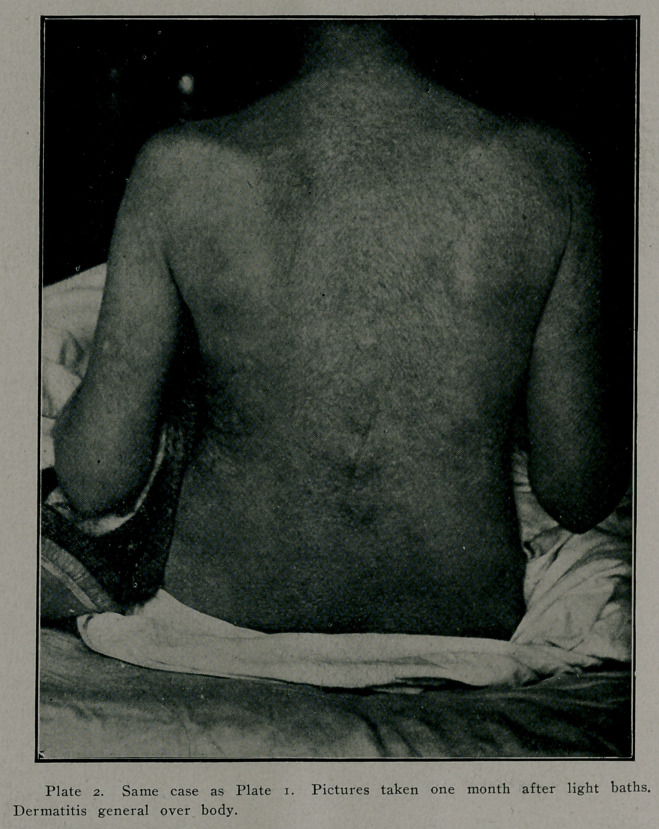# Etiology, Pathology and Treatment of Pellagra

**Published:** 1912-01

**Authors:** Geo. C. Mizell

**Affiliations:** Gastro-Enterologist to Wesley Hospital; Formerly Associate Professor of Physiology and Gastro-Enterology of Atlanta College of Physicians and Surgeons


### Journal-Record of Medicine

Successor to Atlanta Medical and Surgical Journal, Established 1855, and Southern Medical Record, Established 1870.

**OWNED BY THE ATLANTA MEDICAL JOURNAL CO.**

**Published Monthly**

*Official Organ Fulton County Medical Society, State Examining Board, Presbyterian Hospital, Atlanta, Birmingham and Atlantic Railroad Surgeons' Association, Chattahoochee Valley Medical and Surgical Association, Etc.*

EDGAR G. BALLENGER, M. D., Editor.
BERNARD WOLFF, M. D., Supervising Editor.
A. W. STIRLING, M. D., C. M., D. P. H., J. S. HURT, B. Ph., M. D.
GEO. M. NILES, M. D., W. J. LOVE, M. D., (Ala.); Associate Editors.
E. W. ALLEN, Business Manager.

COLLABORATORS

Dr. W. F. WESTMORLAND, General Surgery.
F. W. McRAE, M. D., Abdominal Surgery.
H. F. HARRIS, M. D., Pathology and Bacteriology.
MICHAEL HOKE, M. D., Orthopedic Surgery.
CYRUS W. STRICKLER, M. D., Legal Medicine and Medical Legislation.
E. C. DAVIS, A. B., M. D., Obstetrics.
E. G. JONES, A. B., M. D., Gynecology.
R. T. DORSEY, Jr., B. S. M. D., Medicine.
L. M. GAINES, A. B., M. D., Internal Medicine.
GEO. C. MIZELL, M. D., Diseases of the Stomach and Intestines.
L. B. CLARKE, M. D., Pediatrics.
EDGAR PAULIN, M. D., Opsonic Medicine.
THEODORE TOEPEL, M. D., Mechano Therapy.
R. R. DALY, M. D., Medical Society.
A. W. STIRLING, M. D., etc., Diseases of the Eye, Ear, Nose and Throat.
BERNARD WOLFF, M. D., Diseases of the Skin.
E. G. BALLENGER, M. D., Diseases of the Genito-Urinary Organs.

VOL. LVIII. JANUARY 1912 NO. 10

### ETIOLOGY, PATHOLOGY AND TREATMENT OF PELLAGRA.

BY GEO. C. MIZELL, M. D.

*Gastro-Enterologist to Wesley Hospital; Formerly Associate Professor of Physiology and Gastro-Enterology of Atlanta College of Physicians and Surgeons.*

### PATHOLOGICAL AND PHYSIOLOGICAL CHEMISTRY.

*(Continued from November Journal-Record of Medicine.)*

In the article of November, we followed the fats through the stomach, bowels and blood. The discussion of the athological and physiological chemistry has been limited to the changes which take place in these organs and tissues. It was pointed out how there may be a marked increase in the oxidation of fats when linolin is substituted for olein, palmitin and stearin. The depositing of linolin in adipose tissue shows that at least some of the fat has retained its chemical identity. Not only does the ingested fat show its identity in adipose tissue, but there is reason to believe that this identity is retained by the fats and fatty acids which enter into the various cellular structures.

Lecithins are ester compounds of glycero-phosphoric acid substituted by two fatty acid radicals with a base called choline. The fatty acid radical may be oleic, palmitic or stearic acid, hence the lecthins are of different kinds according to the fatty acid radical. According to Thudicum (Physiological Chemistry, Mandel), two different fatty acid radicals may exist in one molecule of lecithin and according to the same investigator true lecithin always contains one oleic-acid radical.

A thorough study of lecithin from ox heart and muscle has been done by Erlandsen (Mandel). These lecithins, he finds, yield fatty acids that have a higher iodine equivalent than oleic acid, which shows that the fatty acid belongs to the linolic or linolinic acid series. It would be interesting to know the composition of the food of the animals from which these specimens were obtained. The general use of oil seed cake in Europe for feeding will suggest the probability of a food containing linolin. The ingested fat in the ox does not play as important a part in the replacement of the body fat which has been used in metabolism as it does in man.

This statement may be questioned, and in support of it, attention is called to the low per cent. of fat which is present in the food of these animals from which they are able to remain fat and replace the fat that has been used up in metabolism. On the other hand, the human animal, according to Hammarsten, takes on a superfluous amount of fatty tissue when an excess of fat is present in his food, and on partaking of food deficient in fat this accumulation is rapidly exhausted. Herter also states that the ingested fat is more important in this connection in man than in animals.

In the le ithin, or more correctly in the lecithin mixture of the yolk of egg, Cousins find also linolic acid besides the three ordinary fatty acids. The composition of yolk fat is dependent upon the food, as Henriques and Hansen have shown that fat of the food passes into the egg (Mandel).

Thus there may be a proportionate increase in the deposit of ingested fat in man.

Lecithin is a primary constituent of the animal cell and is of great physiological importance. It probably plays a prominent part in osmotic phenomena of cell membrane and in the building up of the complicated nuclein substances of the cell and cell nucleus. Lecithin is especially abundant in brain, nerve, muscle and blood corpuscles. There is a greater proportion of lecithin in the organs of the foetus (brain, liver, heart and muscle) than in the same organs of eight or ten year old children. The child has a store of lecithin which is used up in the first few months of extra-uterine life (Swiestgow, Mandel). There are other compounds of which fats and fatty acids are component parts but which have been less studied than lecithin.

Much attention has been directed to the nervous system of Pellagrins and it is significant that of all tissues except adipose tissue, nerve tissue contains the largest per cent (22%) of fat. The brain is next, with eight per cent. Now, after having pointed out above that linolic acid may occur in lecithin, we may presume that it may enter into other important primary cell structures. It is not assuming too much to suggest that all compounds containing linolic acid necessarily partake of the properties of salts of linolic acid. Thus linolyl-lecythin may be a very unstable compound as compared with stearyl-, oleyl-, or palmityl-lecithin. This unstable property of the fat and cell constituents which contain linolin or linolic acid may account for nervous and mental phenomena and the muscular weakness of linolin consuming animals.

This unstable property favors rapid changes (oxidation) in the important cell constitutents, such as lecithin, protagon and cuorin. In the case of lecithin, because of the part played by osmosis we may have an explanation of the peculiar oedema that occurs in some cases of Pellagra.

The information brought out above may be summed up as follows:

1st. When linolin is consumed it replaces to some extent olein, palmitin and stearin in the fat and adipose tissue of the body.

2nd. The linolic acid of ingested linolin may replace the fatty radicals of olein, palmitin and stearin in the important primary cell structures, such as lecithin, protagon, cuorin, etc.

3rd. Linolyl-lecithin, -protagon, and -cuorin are probably much more unstable compounds than oleyl,- stearyl,- palmityl-lecithin. That this is true is in accord with the unstable constitution of the salts of linolic acid.

The readiness with which linolin and linolic acid compounds undergo oxidation may render them unfit as a staple article of diet. The excessive production of oxidation products which is necessarily favored by a linolin diet may develop a pathological state similar to chronic alcoholism. Herter has pointed out that the fatty acids of the lower fatty acid groups are used up somewhat in the same way as alcohol. It is by no means certain that the oxidation products of alcohol and the incomplete oxidation of fats do not play as great a part in the deleterious action attributed to alcohol, as alcohol itself. (Mild chronic alcohol intoxication is considered a caues of arterio-sclerosis, neuritis, dementia-paralytica, epilepsy, various forms of cirrhosis of the liver, the kidneys (*as in Pellagra*), escape any marked influence. Delirium (*in both conditions*) is usually a result of long-continued action of the poison on the brain.

As a tissue poison, the effects of alcohol are seen on parenchyma, particularly epithelium and nerve, producing a slow degeneration. It inhibits oxidation by being oxidized in place of fat, thereby producing a general condition of steatosis. Forgettia may supervene (*Osler.*) The action of alcohol as a poison and the action of the poison causing Pellagra are very similar in many respects. Tanzi gives the pathological anatomy of the nervous system in Pellagra and calls attention to its similarity to that of alcoholism. Especially does he mention the changes present in acute pellagra and the occurrence of the same lesions in delirium tremens.

It is a matter of note that the delirium of alcoholism often does not appear until after the use of alcohol is discontinued. It may be significant that incomplete oxidation of fats and fatty degeneration are some of the chief pathological states prevailing in alcoholic intoxication.

Thus, in linolin, we have a product that may not be injurious when consumed within certain limits and as in alcohol may be tolerated in larger quantities by one organism than by another. In making this comparison it must be borne in mind that linolin may be stored in the body in such quantities that it is months in being removed, while the poisonous effect of alcohol is more or less limited to the time of consumption.

EFFECT OF SUNLIGHT.

The relation of the sunlight to the skin lesions in Pellagra has been recognized for a long time. Most investigators have noted that the erythema is limited to the exposed surfaces. It has also been noted that the elbows and knees show lesions even when not exposed, but this is not peculiar to Pellagra. Drs. Babcock and Lavender attribute this to pressure, which no doubt plays a part, as we will see later. Also there is often a dermatitis in those regions that come in contact with irritating discharges, or where two surfaces oppose, as around the vulva and scrotum. The above lesions should not be looked upon as typical of pellagrous dermatitis. The lesions due to irritating discharges rapidly return to normal when protected by a bland ointment, while the contrary is the case with the typical dermatitis.

Dr. Woodruff (The Effects of Tropical Light upon White Man), notes symmetrical skin lesions occurring on the elbows and knees, which he attributed to vaso-motor disturbance. It has been asked by Drs. Babcock and Lavinder and others if the skin lesions may not be of nervous origin. When it is observed that these lesions do not occur except on the exposed parts; that they disappear when protected from the light to recur again upon exposure and that they occur over the entire body when it is exposed, it is hard to see how we are to escape the conclusion that the primary cause is the existence in the skin of some substance in or under the skin, that develops into or causes a protoplasm poison when exposed to the peculiar chemical action of the sun. Another fact which discredits the idea of nervous origin is that the changes in the nervous system are in no wise peculiar to pellagra.

The brain and spinal cord in chronic cases present changes which also occur in chronic alcoholism and amæntia. These changes seem to be peculiar to psychoses such as amentia and alcoholism. It is worthy to note that all such lesions have their origin in disturbances of nutrition that are caused by intoxication. Thus, in pellagra, alcoholism and amentia, the lesions in the brains and cord are found in the same region. The peripheral neuritis of alcoholism shows a predilection for the peroneal nerve (*Tanzi*) which also seems to be the case in pellagra. The lesions of alcoholism and amentia are not accompanied by dermatitis.

The symmetrical bilateral occurrence of the skin lesions has been set forth as an evidence that they are of central nervous origin. When it is considered that exposure is symmetrical and that when the whole body is symmetrically exposed the dermatitis occurs all over the body, it is hard to see wny the nervous system should be held responsible. The accompanying photograph is of a patient now under observation. She was exposed to the sun, clad in a thin night dress for three weeks. When exposure began there was no dermatitis. In four weeks the hands were severely affected and desquamation was complete one month later when the dermatitis on the body was at its height. At this time the photograph was taken. The patient has lost the most of her heavy suit of hair and the dermatitis on the feet is more severe than on the body. The diagnosis in this case was made by Drs. Harris and Patillo before I was called in, so there is no question as to diagnosis. As mentioned, the distribution of linolin in the body may account for the difference in the degree of dermatitis on the extremities and on the body when there is complete exposure. It should be clear that the sunlight is the secondary or precipitating cause of the dermatitis.

The question naturally arises as to how the sunlight induces the dermatitis. As will be shown later, light accelerates oxidation and its penetrating power is sufficient to reach the subcutaneous tissues and here is no reason why this influence should not be effective on subcutaneous and cutaneous constituents. It is necessary to recall here the demonstration of Cloes, as follows: The changes (oxidation) in fats and oils are most rapid under white light, less under blue and much less under red, yellow and least of all under green. While the various lights vary in rate as to their effect, if exposure is continued long enough, the final result is about the same. The source of oxygen necessary may be the blood, or it may be supplied by cutaneous respiration, which amounts to about one-fortieth to one-fiftieth of the respiratory function.

Anticipating that it may be asked why the suspected end-products have not been identified by chemical analysis and thus prove without argument the correctness of these deductions, it only needs to be pointed out that there are many ideas concerning the chemistry of fats that are accepted which are not capable of chemical demonstration. For example, chemists have no means by which to determine so important a change as the rancidity of fats and point to so crude a test as the sense of smell as the best method of detecting rancid fat. By applying this method in a severe case of dermatitis, especially where desquamation is in large scales, one cannot fail to detect a rancid odor. Patients themselves have often detected this odor upon their hands and also emanating from their bodies in some cases.

The constitutional effects of sunlight, while not definitely understood have been recognized for some time. Recent reports by eminent investigators bring to light interesting information, and we may expect the points of difference to be cleared up in the near future. For our present purpose, it is not material whether the long or the short waves of energy bring about pigmentation, or which is responsible for the chemical action. We will take a position which is not questioned, viz., that the effect of the energy emanating from the sun, whether infra-red, light, or actinic, favors oxidation according to the intensity and the length of period of exposure. As direct evidence of this, attention is again directed to the demonstration of Cloes and the various works on fats and oils as to the influence of light in promoting oxidation of fats.

These results, of course, took place outside of the body. However, a condition exists in the body which further favors oxidation. That is, when fat comes in contact with the alkaline

Plate 1. Atlanta Case. Dry scaly dermatitis appearing on body after three weeks' exposure to strong daylight and sunlight. Patient clad in light night-dress during exposure.

blood containing oxygen we have a condition similar to fat in a solution of alkaline permanganate or peroxid. The above conditions obtaining, the action of sunlight in each case should be the same, differing only in degree according to the penetration of the body by the light.

Where the sunlight falls upon a body it is resolved into three parts; one part is reflected and does not enter the body at all. The second part is absorbed, that is, converted into another form of energy which may be either heat, chemical or electric energy. The third part is transmitted, that is, passes through the body if it is not too thick. The human body which is too thick to transmit any of the sun's ray is affected by all the rays that are not reflected. The result of exposure to sunlight is the sum total of the action of all rays which fall upon the body except those that are reflected. The extent of reflection or absorption of the rays by a human body is entirely governed by the color of the skin and the intensity of the energy.

Major Chas. E. Woodruff, in his book "The Effects of Tropical Light on White Men," has clearly shown that sunlight is deleterious to all men who are not protected by sufficient pigment. There may be some question as to the mode of action of the sun's rays, but the statistical data is sufficient to prove his contention. Major Woodruff holds that pigment is evolved for the purpose of protection of the body against the chemical (actinic) rays.

According to his argument, the inhabitants of the tropics after numerous generations finally become dark-skinned or extinct. The death of white men in the tropics is the result of the stimulating effect of actinic rays upon protoplasm. This stimulation, he holds, increases atomic and molecular motion to the point of dissolution. This increased atomic and molecular motion, he believes, is imparted by the vibration from actinic rays or waves.

Major Woodruff is probably correct in his ideas and argument, but he may not have entirely covered the pathological chemistry of the action of sun energy. We may get some idea of the pathological chemistry by a study of the physiological action of the different wave lengths of energy emanating from the sun.

The local and constitutional effects of sunlight or artificial light upon human tissue are complex and are not always eas'ly understood, the metabolic processes in any case be:ng very difficult of demonstration. That light increases oxidation is one of the clearly recognized effects and is the action that is v'tal to

Plate 2. Same case as Plate 1. Pictures taken one month after light baths. Dermatitis general over body.

our subject. The chem:ical action of light in excess, by its oxidation effects, are injurious to human cells and certain forms of germ life. The actin'c effect of the higher frequencies, and the combined action of all the frequencies of light and heat, have been demonstrated to be capable of increasing the oxidation processes. This effect is greatest under the higher frequencies of the radiant energy from the sun and the hyperaemia from exposure to these rays is intensely irritating. Tanning, which is caused by sunlight, is due to actinic rays and is a natural protection of the organism against these rays. Pigment is more or less opaque to all frequencies. The blood is somewhat opaque to the actinic rays, hence they do not penetrate very deep. This property of blood is dependent upon the presence of haemoglobin, which no doubt is acted upon, giving rise to the tanning.

Neils Finsen has demonstrated that when the tissues are rendered anaemic by pressure the ultra-violet waves are more penertating. The infra-red radiations have greater pentrating powers than the higher light frequencies, blue, indigo and violet. This rule applies until the X-rays are reached and appears to have some connection with the relation of the wave length to the atomic weight. The penetrating powers decrease up to the X-rays, which go straight on. At this point the effect upon tissue seems to change. Thus the action of long or frequent exposure to X-rays inhibit metabolic process, while the effect of the lower frequencies stimulate.

It has been shown that the sun's rays penetrate to considerable extent and with sufficient degree of intensity to produce marked effect upon the local oxidation process. This action is evident in sunburn and tanning. Further proof is the occurrence of sun-stroke. In these conditions it is seen that the cell constituents and superficial tissues may be profoundly affected unless protected. It is then evident that in some cases the sunlight may stimulate oxidation of the fat present in the skin and subcutaneous tissue:

The above has been set forth in explanation of the skin lesions which occur in Pellagra, but if the climatic influences were limited to these lesions much in the periodic exacerbations of the constitutional symptoms would be left unaccounted for.

Not only does sunlight produce pathological conditions, as evidenced by tanning and sunburn, but it may greatly influence general metabolism. Acute effects are manifested by sun-stroke and milder pathological conditions developing in white inhabitants in the tropics have received much attention from the Medical Officers on duty in the tropical regions.

Dr. Woodruff offers much evidence to show that in order to retain health even in such climates as the United States, excepting the northern tier of States, it is necessary for the white race to take some precautions against the deleterious action of sunlight. This deleterious effect is attributed to the stimulating action of light which property is made use of as a treapeutic agent. The stimulating and tonic effects of radiant light and heat upon general metabolism according to Dr. Snow, in "Radiant Light and Heat and Convective Heat," are due to several specific actions or effects induced on the tissues in the circulating fluids of the body.

1. *"The Action on the Blood.*—(1.) The oxidizing influence of radiant light and heat favor to a remarkable degree active tissue metabolism. (2). The oxygen carrying function of the blood is enriched by an increased percentage of hemoglobin due to the direct action of light rays, and (3). The lymphatic are rendered more active in eliminating waste products and toxins by the sweat glands and other emunctories of the body.

2.—*"The Superficial End-Organs* are stimulated to a greater activity with an increased tissue change, both anabolic and catabolic.

3.—*The Deep Spinal Centers* are reflexly stimulated to greater reflex activity by the intense effects of the application of radiant light and heat to the peripheral neurones, thereby arousing greater general activity of the vital centers, particularly the perspiratory, cardiac, and excretory centers.

4.—*"The General Diffusion of Heat* which takes place by convection from the blood heated at the periphery, promotes general tissue oxidation and elimination throughout the organism.

5.—*"The Actinic and Thermic Action* of the radiant light and heat upon the germs in local areas of infection, causes inhibition of activity and destruction of the germs by the phagocytes, hereby relieving the tissue generally from the toxic materials otherwise thrown out.

6.—"*The Induction of Superficial Hyperemia,* local or general, promotes nutrition in the tissues by an increase of nutritious pabulum distributed throlgh the tissues, as well as an increase in the number of nature's scavengers, the phagocytes, where hyperemia exists, thereby increasing the general tissue resistance, as well as a a greater metabolic activity.

7.—"*The Stimulation of Increased Elimination* through the sweat glands and other emunctories, induces the removal from the system of the poisonous toxins which vitiate the general system and cause general impairment of metabolism.

"The action thus instituted by the systematic employment of radiant light and heat, it will be readily seen, are most valuable; because, when administered in a technically scientific manner with the requisite attention to the details of administration, they favor the elimination of waste materials, and the increased activity of general and local metabolism with marked increase of oxidation and tissue combustion, generally favorable to the establishment of the normal functions of the body. The increase of superficial hyperaemia not only influences the insitution of increased local tissue resistance, but in conditions of impaired kidney functions and arterio-sclerosis, relieves arterial tension and facilitates the elimination of the waste products through the skin, which the impaired kidneys are unable to eliminate.

The above, as will be seen, is Dr. Snow's interpretation of artificial radiant light and heat as a therapeutic agent. However, he has previously stated that sunlight furnishes all that could be desired in light therapy, but for obvious reasons is variable and not always accessible.

When it is considered that the energy from the sun is many times more intense than any artificial light that can be produced, we see that it it much more potent in its action.

That the energy of the sun is of vast importance to health and germacidal in its effects is recognized, at the same time such a powerful force by its stimulating and chemical efforts becomes harmful from too frequent or prolonged exposure.

The injurious effects of light energy are in part if not altogether explained by the increased chemical action (oxidation) induced in the tissue.

As evidence that this is true, the following is quoted from Light Energy: Cleaves.

"*Light Energy in Relation to Metabolism.*—In our study of the action of light energy upon animal organisms it was found that after an initial decline there was an increase in weight of the animals exposed to light as against those kept in darkness and that also tissue change went on more rapidly under the influence of light. This action may be explained by a stimulation of the nervous system, which in its reaction stimulates other vital functions, or it may be a direct action upon the blood stream itself. Of the fact that certain modifications in the tissue change in both men and animals take place under the influence of light energy, there is no question. This action must be twofold, *i. e.,* upon the blood directly, and indirectly through the nerve system.

"There are certain observations based upon experimental work which seem to favor the theory of a stimulating action upon the nervous system. Quincke demonstrated by his experiments that various tissue cells, pus, blood, muscle, kidneys, liver, etc., absorbed more oxygen in the light than in the dark. So long as they are not quite dead severed muscles and nerves, according to Moleschott and Fubini, eliminate carbonic acid more freely in the light than in the dark.

"That light energy influences the oxidation of the tissues is the concensus of opinion, and the author believes that this is largely due to a direct action upon the blood itself. Ultra-violet and blue-violet frequencies are absorbed by the blood better than by any other tissue. Physically it seems quite possible that the ultra-violet frequencies are in step or tune, so to speak, with the vibrational activity of the oxygen atom, in other words, that there is sympathetic resonance between them.

"The observations of many experimenters on many different occasions tend to show that on both men and animals light energy has an influence tending to increase the oxygenating power of the blood and the oxidation of the tissues.

"From the chemical point of view the bactericidal power of light energy is a prenomenon of oxidation. For its successful action the presence of oxygen is necessary.

"The experiments of Finsen show that ultra-violet rays act as a vigorous irritant to the nerve system and that by them skin reflexes are increased. From the nature of their action upon the skin (chemical), such an increase of the activity of skin reflexes and irritation of the nerve system would rationally follow.

"That there is then an effect from light energy upon the living organism is shown: (1.) By its irritant effect upon the skin, intense light producing inflammation. (2.) By its action upon the sweat glands, promoting perspiration, this is true of chemical light energy as well as of thermal. (3.) By its direct action upon the blood and the blood vessels, dilatation. (4.) By exposure of large superficial areas of the body to the action of intense light energy there results an increased amount of blood in the superficial vessels and a depletion of the internal organs or viscera. (5.) By a direct or indirect influence light energy modifies the transmutation of matter. (6.) By the action of light energy in relation to motor excitation. (7.) By its parastiticidal action. (8.) An excess of light stimulus (in common with too great an expenditure of any energy), is destructive and paralyzing. By its dermatitis, a prolonged erythema with tendency to recurrence and insolation are produced.

### Color of Skin.

It has been pointed out that exposure to sunlight causes pigmentation and that this property is due to actinic rays and also that the presence of yellow, brown or black pigment in the skin renders the skin more or less opaque to all frequencies.

In describing the dermatitis of Pellagra the pigmentation is especially noted and referred to in a mysterious manner, whereas it presents nothing peculiar or different from varying degrees of sunburn. Perhaps what has attracted this attention is that the pigmentation is removed with desquamation of the dead epithelium. The pigmentation continues to persist if the patient is continually exposed. Pellagrins who are confined to the house will retain a normal skin, or rather the skin tends to bleach and become anaemic in appearance. It is possible that this factor alone accounts for the comparative freedom of the negro from the dermatitis. The only statistics in hand showing the proportion of negroes to white is found in the report by the special commission selected by the Tennessee State Board of Health, which shows 18 negroes in a total of 316 cases.

Color of the skin may also account for the predisposition of certain members of a family to develop the disease more rapidly than others. In many instances among my cases the blondes have developed the disease first and the brunettes seem to be somwhat resistant. Pigmentation and protection from light will be again referred to, later.

It must be borne in mind that the sunlight may penetrate even the blackest skins and when it is sufficiently intense will cause a severe dermatitis in these skins. In an effort to establish a prodromal period, and therefore a resemblance to infectious diseases writers have noted that constitutional symptoms become less severe with the appearance of the dermatitis. This is often, not always, true, but there is no causative relation between the improvement and the appearance of the dermatitis. The real reason is found in the fact that the dermatitis does not appear until several days after the exposure which is the exciting cause of the dermatitis and the constitutional symptoms. Beginning with and for an indefinite length of time after the exposure the patient may be ill enough to confine him to the house, in which case his symptoms will somewhat subside about the time the dermatitis appears.

If this view of the disease is correct there is a progressive pathological state which continues independent of climatic influences. In some cases there may be apparent complete recovery in the winter, but with the onset of clear, hot days each succeeding outbreak is more severe and finally the symptoms, except the dermatitis, persist through the winter. It is easy to conceive of quite a general prevalence of this disease without recognition, when the climate does not meet the requirements for the development of the skin lesions.

Thus we see that metabolic rpocesses with tendency to exhaustion are increased by the sunlight and heat of summer. There is reason to believe that this in part accounts for the exacerbations in gastro-intestinal and other disorders that occur in this climate during the summer. We can see, then, how there may be a pathological state from consuming linolin in progress the whole year round. This constitutional condition will be increased during the hot bright summer of such regions as Georgia, South Carolina, Mississippi, Texas and Illinois. Exceptional season may greatly modify the development of the disease and in these factors we have an explanation of the comparative freedom of certain regions. As far as I am able to ascertain from the known geographical distribution of Pellagra, it is limited almost entirely to the belt of winter rains and dry summers. There are apparent exceptions to this rule, which are determined by local climatic conditions. One of the points advanced by supporters of the maize theory is that a wet season is often followed by an outbreak of Pellagra, which may be true not only for the reason that the corn was spoiled through moisture, therefore unhealthy, and by interfering with digestion acted as a secondary cause, but also that a precipitation of the watery vapor cleared the sky and allowed the sun to shine on the laborer who goes to work in the fields.

There has been no suggestion offered by the writers on the effects of light as to specific chemical changes induced in epithelium, nerve, muscle, blood, etc., except those that may be included in the general terms increased activity and oxidation. This increased oxidation, it appears, is a spontaneous combustion independent of the metabolic directing forces and in excess of the needs of the body. Direct evidence of increased oxidation by light of any certain cellular constituent or tissue is confined to the fats and fatty acid salts. We have seen how light greatly accelerates oxidation of the fats and oils. We have seen that light penetrates human tissue with sufficient intensity to increase oxidation and produce marked local pathological conditions, even resulting in death of tissue (sunburn) and under some conditions produce constitutional symptoms and death (sunstroke).

Then may we not hold that the oxidation of fats in living tissue is increased under the influence of the sun's rays? And, furthermore, why should the fats oxidized under the above influences not give rise to the same oxidation products as those that are produced outside of the body?

401 Empire-Life Building.

## Figures and Tables

**Plate 1. f1:**
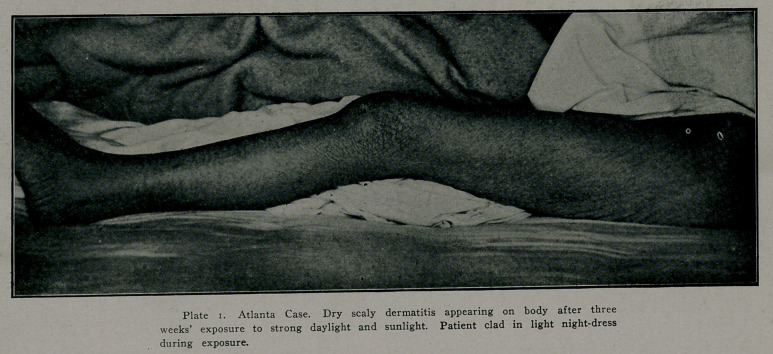


**Plate 2. f2:**